# Geographical traceability of gelatin in China using stable isotope ratio analysis

**DOI:** 10.3389/fnut.2023.1116049

**Published:** 2023-02-16

**Authors:** Shuang Li, Di Jiang, Jinglin Li, Yuhua Ma, Jian Yao, Lin Du, Yisheng Xu, Yuan Qian

**Affiliations:** ^1^State Key Laboratory of Chemical Engineering, East China University of Science and Technology, Shanghai, China; ^2^Department of Molten Salt Chemistry and Engineering, Shanghai Institute of Applied Physics, Chinese Academy of Sciences, Shanghai, China; ^3^Department of Tritium Science and Engineering, Shanghai Institute of Applied Physics, Chinese Academy of Sciences, Shanghai, China

**Keywords:** stable isotope ratios, gelatin, geographical traceability, bone, processing

## Abstract

Geographical traceability is crucial to the quality and safety control of gelatin. However, currently, methods for gelatin traceability have not been established anywhere in the world. This study aimed to investigate the possibility of differentiating the geographical origins of gelatin from different regions in China using stable isotope technology. To achieve this objective, 47 bovine stick bone samples from three different regions (Inner Mongolia, Shandong, and Guangxi, respectively) in China were collected, and gelatin was extracted from these bones using the enzymatic method. The fingerprint characteristics of stable isotopes of δ^13^C, δ^15^N, and δ^2^H of gelatin from different regions in China were studied. Moreover, isotopic changes from the bone to gelatin during the processing were examined to evaluate the effectiveness of these factors as origin indicators. The results of the one-way analysis of variance (ANOVA) showed that the δ^13^C, δ^15^N, and δ^2^H of gelatin from different regions display significant differences, and using the linear discriminant analysis (LDA), the correct differentiation of origin reached 97.9%. Certain differences in stable isotope ratios were observed during the processing of bone to gelatin samples. Nonetheless, the fractionation effect caused by the processing of bone to gelatin samples was not sufficient to influence the identification of gelatin from different origins, which proves that δ^13^C, δ^15^N, and δ^2^H are effective origin indicators of gelatin. In conclusion, the stable isotope ratio analysis combined with the chemometric analysis can be used as a reliable tool for identifying gelatin traceability.

## 1. Introduction

Gelatin, a natural polymer extracted from the bones, skin, or connective tissue of animals, has been widely used in food, pharmaceuticals, cosmetics, and other industries due to its distinctive physicochemical property (1, 2). In recent years, a variety of gelatin safety incidents, such as animal epidemics, mislabeling, and adulteration, have occurred frequently, and consumers have increasingly focused on the origins of gelatin. Thus, laws and regulations were promulgated in many countries to regulate the sources of gelatin (3). However, related studies on the methods differentiating the origins of gelatin from different regions are limited at present. It is difficult for the government to ensure effective supervision of the source of gelatin, which not only greatly compromises public health and life safety but also highly restricts the development of the gelatin industry. Therefore, an effective method is urgently required to trace the geographical origins of gelatin.

Recently, various methods, such as stable isotope ratio analysis (4, 5), mineral element analysis (6), fatty acid analysis (7), near-infrared spectroscopy (NIRS) (8–10), high-performance liquid chromatography (HPLC) (11), mass spectroscopy (MS) (12), and DNA-based technology (13, 14), have been applied to origin traceability and authentication of animal-derived products (15). However, these methods have a few shortcomings. For instance, the processing cost and experimental operation requirements of mineral element analysis are high. The sample pretreatment time for fatty acid analysis is prolonged, and is easily affected by feed, genetics, variety, and processing. The sensitivity of near-infrared spectroscopy is not high, and its data processing is difficult. HPLC has a limited capacity for qualitative determination and makes considerable use of hazardous solvents that are toxic to human health. The DNA-based technique requires numerous samples, and it is challenging to select effective molecular markers. At present, stable isotope ratio analysis is considered a more accurate and quicker method and has become the most commonly used technology for validating the traceability and authenticity of animal-derived foods. Geographical origins of some animal-derived products, such as meat, dairy products, seafood, and honey, were successfully traced using stable isotope ratio analysis (16–19). Meanwhile, research on the transformation law of stable isotopes from raw material to the terminal product during processing has also been carried out (20–22), which can further provide theoretical support for animal-derived product traceability.

Many studies on gelatin focused on species identification, and there are few related studies on traceability methods for gelatin are available at present. Jannat et al. successfully differentiated bovine, porcine, and fish sources of gelatin in commercially pure gelatin and gelatin-containing food and drug products using real-time polymerase chain reaction (PCR) and the analysis of mass spectrometry (MS)-based proteomic datasets (23). Sha et al. successfully identified fish gelatins in seven commercial cyprinid fishes using high-performance liquid chromatography (HPLC) and high-resolution mass spectrometry (HRMS) (24). Cai et al. presented a new strategy for the simultaneous rapid identification and quantification of gelatins from various species using ultrasound-assisted digestion-ultra-high performance liquid chromatography-tandem mass spectrometry (UPLC-MS/MS) (25). In our previous study, Jiang et al. successfully traced the origin of bone materials of gelatin in China using stable isotope analysis and mineral element analysis (26).

Based on the aforementioned studies, stable isotope ratio analysis coupled with chemometric analysis was first used to trace the geographical origin of gelatin in this study. The key aim of this study was to explore the regional differences in stable isotopes in gelatin from China and develop a reliable identification method. In addition, changes in δ^13^C, δ^15^N, and δ^2^H from bone raw materials to gelatin during processing were also investigated to evaluate their effectiveness as geographical origin indicators. The results of the present study will provide a new idea and strategy for the origin traceability of gelatin.

## 2. Materials and methods

### 2.1. Sample information

A total of 47 bovine stick bone samples were sampled from three representative regions of China, including Mashan County of Guangxi Province (GX), Heze City of Shandong Province (SD), and Hulun Buir City of Inner Mongolia (NMG) (Table 1). Defatted bovine granules (d <20 mm) were obtained using the conventional method (26) and then stored in a dryer.

**Table 1:** Region-wise information of bovine stick bone samples.

**Region**	**Longitude and latitude**	**Sampling time**	**No.of samples**
Mashan County of Guangxi Province (GX)	108°17′E, 23°72′N	November 2018	16
Hulun Buir City of Inner Mongolia (NMG)	115°31′-126°04′E, 47°05′-53°20′N	December 2018	15
Heze City of Shandong Province (SD)	114°45′-116°25′E, 34°39′-35°52′N	January 2019	16

GX, SD, and NMG are the abbreviations of Guangxi, Shandong, and Inner Mongolia, respectively.

### 2.2. Experimental methods

#### 2.2.1. Bone gelatin extraction

**Figure 1:**
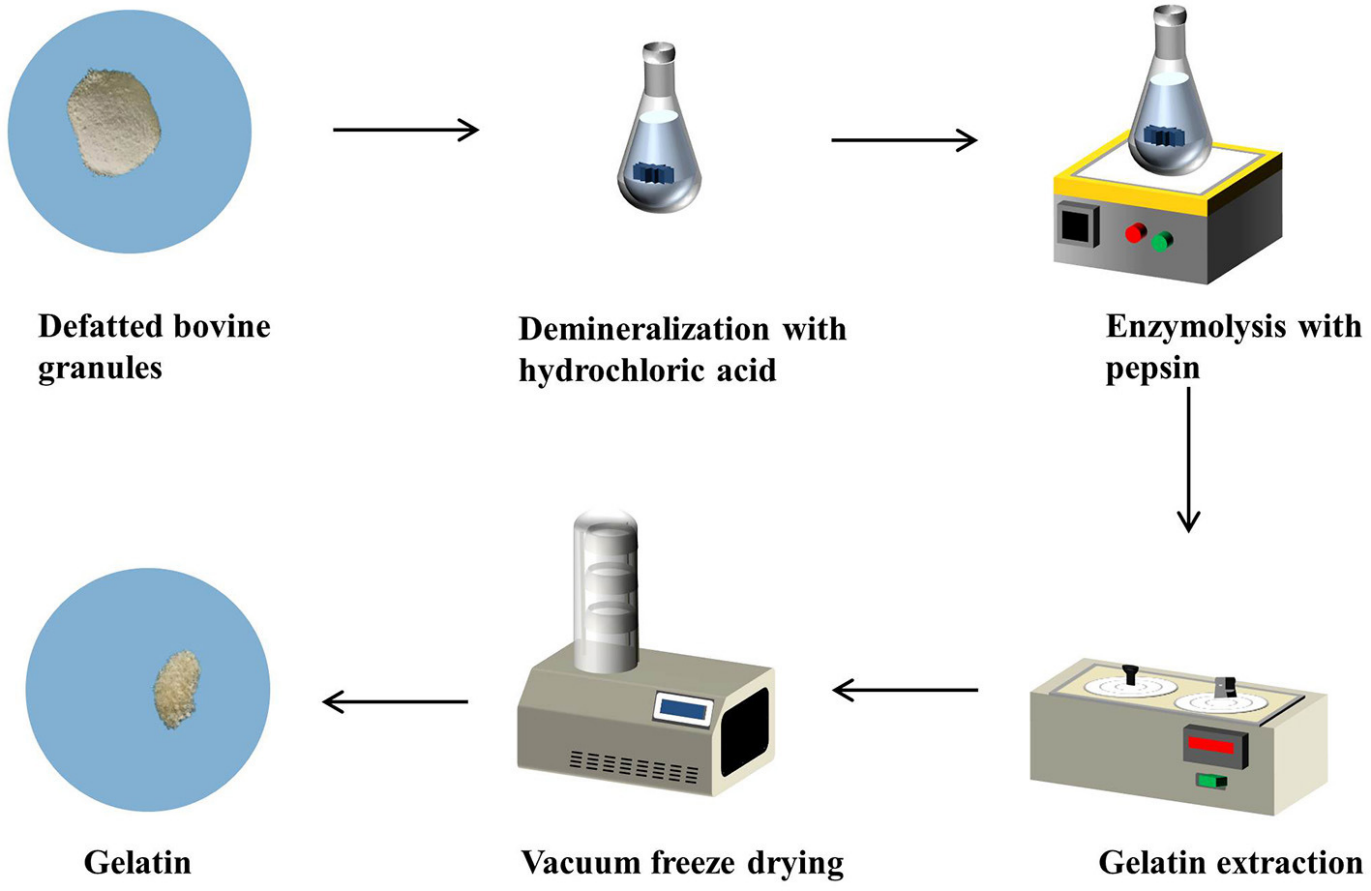
A flowsheet of gelatin extraction by the enzymatic method.

Gelatin was extracted from defatted bovine granules using the enzymatic method (27), as shown in Figure 1. Defatted bovine granules were soaked in 1 M hydrochloric acid (HCl) (Sinopharm Chemical Reagent Co., Ltd., Shanghai, China) at 25°C for 3 h with continuous shaking at the solid-to-solvent ratio of 1:9 (w/v). The mixture was washed until a neutral or faintly basic mixture was obtained and dried at 65°C for 12 h to obtain defatted demineralized bovine granules. Then, the defatted demineralized bovine granules were mixed with pepsin at 40 U/g, deionized water (Millipore Milli-Q Advantage A10 Water Purification System, Millipore, USA) was added at the ratio of 1:9 (w/v) and adjusted to pH 2, and then the mixture was stirred for 3 h at 25°C. Subsequently, the mixture was washed with deionized water and adjusted to pH 5 to ensure that no pepsin remained in the mixture. Later, the pretreated bovine granules were mixed with deionized water at a ratio of 1:2 (w/v) and adjusted to pH 5 to extract the gelatin at 60°C for 3 h with continuous stirring; the gelatin extracted solution was obtained through centrifugation. Finally, the obtained solution was dried to 5 mL at 50°C in an oven (DHG-9140 A, Shanghai Huitai Instrument Manufacturing Co., Ltd., Shanghai, China) and vacuum freeze-dried (HXLG-10-50B, Shanghai Huxi Industrial Co., Ltd., Shanghai, China) for 3 days to obtain solid gelatin samples.

#### 2.2.2. Characterization of bovine gelatin

##### 2.2.2.1. Ultraviolet (UV) spectrum

Ultraviolet spectra of gelatin samples were obtained using a Hitachi U-3900 spectrophotometer (Hitachi Co., Ltd., China). Gelatin samples were dissolved in 0.5 M acetic acid to form a mixed solution at room temperature. The spectra were obtained at a resolution of 4 cm^−1^, and the measurement range varied between 800 and 200 cm^−1^.

##### 2.2.2.2. Fourier transform infrared (FTIR) spectra

The FTIR spectrum (Bruker Optics TENSOR 27 FT-IR Spectrometer, Bruker Optics Inc., Billerica, MA, USA) of gelatin samples was recorded using the tablet method. Briefly, the bovine gelatin samples were mixed and ground with potassium bromide (KBr) in the ratio of 1:100 to make a tablet; the spectra were obtained at a resolution of 4 cm^−1^ and the measurement range varied between 400 and 4,000 cm^−1^. Automatic signals were collected in 32 scans at a resolution of 4 cm^−1^.

#### 2.2.3. Stable isotope ratio analysis

Stable isotope ratios were determined using an Elemental Analyzer (EA) (Flash 2000 HT, Thermo Fisher Scientific, USA) connected to an Isotope Ratio Mass Spectrometer (IRMS) (Delta V MAT 253, Thermo Fisher Scientific, USA). IAEA-600 (δ^13^C = −27.771 ± 0.043‰ VPDB, δ^15^N = 1.0 ± 0.2‰ air N_2_), USGS24 (δ^13^C_VPDB_ = −16.05 ± 0.07‰), USGS42 (δ^15^N_air_ = 8.05 ± 0.10‰, δ^2^H_VSMOW_ = −72.9 ± 2.2‰), and USGS43 (δ^2^H_VSMOW_ = −44.4 ± 2.0‰) were performed to calibrate the stable isotope data.

For the δ^13^C and δ^15^N analysis, appropriate amounts of gelatin samples were weighed into tin capsules and placed into the EA using an autosampler. Carbon and nitrogen in the sample were converted into carbon dioxide (CO_2_) and nitrogen gas (N_2_) in an oxidation–reduction furnace at 980°C, and then, the gases were separated using the gas chromatography (GC) before being sent to the IRMS for analysis.

For the δ^2^H analysis, appropriate amounts of gelatin samples were weighed into silver capsules and introduced into the EA using the autosampler. Hydrogen in the sample was converted into molecular hydrogen gas (H_2_) in a pyrolysis furnace at 1,380°C, and then, the gases were separated using the GC before being sent to the IRMS for analysis.

Stable isotopic ratios (^13^C/^12^C, ^15^N/^14^N, and ^2^H/^1^H) were expressed in δ notation in parts per thousands (‰) and were calculated using the following equation:


(1) $$\delta \;‰=\frac{R_{sample}-R_{standard}}{R_{standard}}\times \;1000,$$

where ***R_sample_*** is the isotope ratio of the sample and ***R_standard_*** is the isotope ratio of the international reference material.

### 2.3. Statistical analysis

The data were analyzed using SPSS 24.0. A *post-hoc* Duncan's test of a one-way analysis of variance (ANOVA) was performed to determine significant differences between the mean values of gelatin samples from different regions (*p* < 0.05) (28). A paired sample *t*-test and Pearson's correlation analysis were, respectively, used to analyze the variability and correlation in δ^13^C, δ^15^N, and δ^2^H from bone to gelatin samples during processing to evaluate the effectiveness of these factors as geographical origin indicators (*p* < 0.01) (20, 29). The linear discriminant analysis (LDA) was used to establish a classification model, and its performance was assessed using cross-validation (30).

## 3. Results and discussion

### 3.1. UV spectra

**Figure 2:**
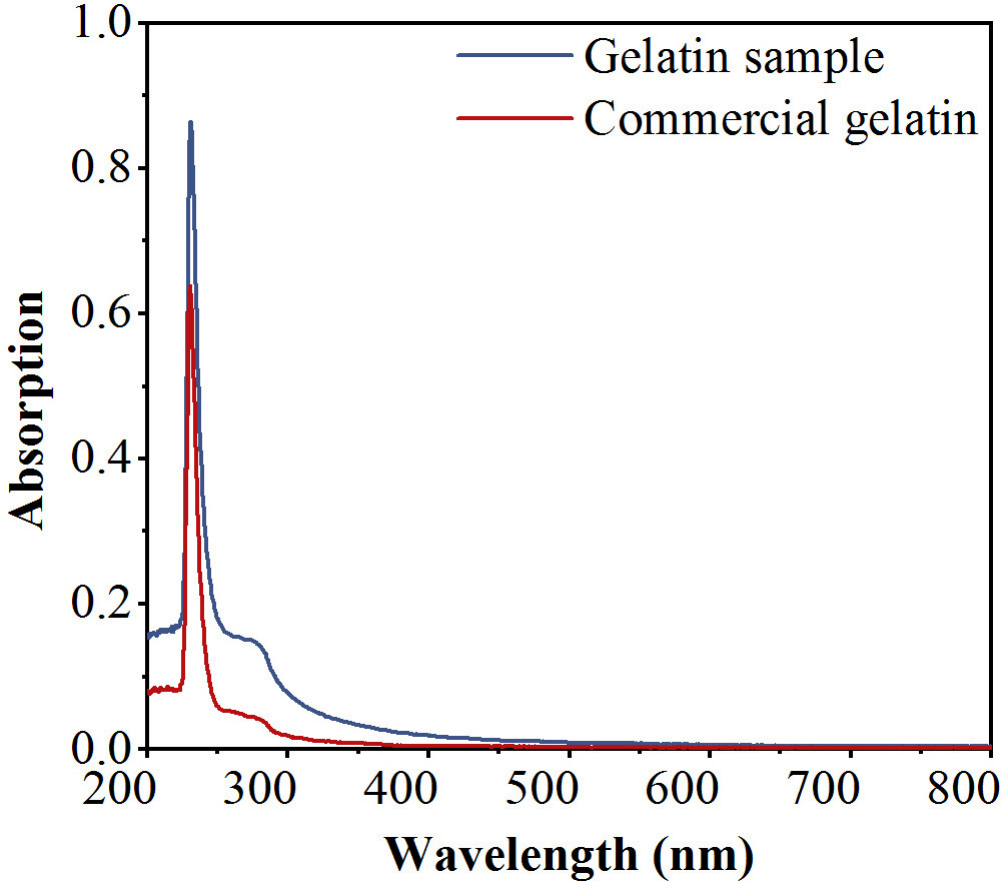
Ultraviolet (UV) spectra of the gelatin sample (GS) and commercial gelatin (CG).

The UV spectra of the gelatin sample (GS) and commercial gelatin (CG) (Shanghai Titan Technology Co., Ltd., Shanghai, China) are depicted in Figure 2. The GS is highly similar to the CG in the spectra. The absorption peak was recorded at 220–240 nm and was mostly associated with the presence of peptide bonds in the polypeptide chains of gelatin. Compared to CG, GS has a small hump at 270–280 nm, which may have been caused by a few aromatic residues, such as phenylalanine, tyrosine, and tryptophan (31). As the two substances show similar spectra, GS is likely to be gelatin.

### 3.2. FTIR spectra

**Figure 3:**
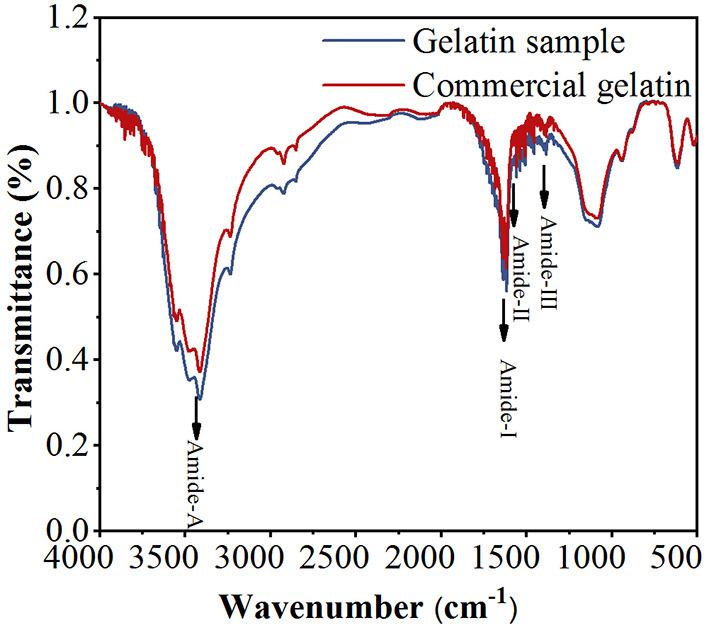
Fourier transform infrared (FTIR) spectra of the gelatin sample (GS) and commercial gelatin (CG).

The FTIR spectra were used to analyze the functional groups and secondary structure of gelatin. As shown in Figure 3, both GS and CG show four infrared (IR) peaks in their spectra, representing amide-A, amide-I, amide-II, and amide-III, respectively. The amide-A band is associated with the NH stretching vibration coupled with hydrogen bonding at the wavenumbers of 3,400–3,440 cm^−1^ (32). The amide-I band is assigned to the C=O stretching vibration coupled to contributions from the CN stretch, CCN deformation, and in-plane NH bending modes, ranging from 1,600 to 1,700 cm^−1^ (33). The amide-II band is attributed to the combination of the NH in-plane bend and the CN stretching vibration in the range of 1,500–1,560 cm^−1^ (34). The amide-III band appears at 1,200–1,300 cm^−1^, due to CN stretching, NH in-plane bending, and wagging vibrations from the CH_2_ groups (35). Amide-A, amide-I, amide-II, and amide-III of GS and CG are found at 3,415.76, 3,415.76, 1,617.23, 1,617.23, 1,559.37, 1,558.41, 1,384.82, 1,385.79 cm^−1^, respectively. Amide-III of GS and CG is shifted to a higher wavenumber, indicating an increased random coil or disordered structure in gelatin (32). Similar peak locations appear for both GS and CG within the reasonable wavenumber range of these peaks, and the striking similarity of the GS and CG peak locations suggests that their secondary structures are similar. Combined with the UV spectra, it can be considered that the GS extracted from defatted bovine granules is gelatin.

### 3.3. Stable isotope ratio analysis

**Figure 4:**
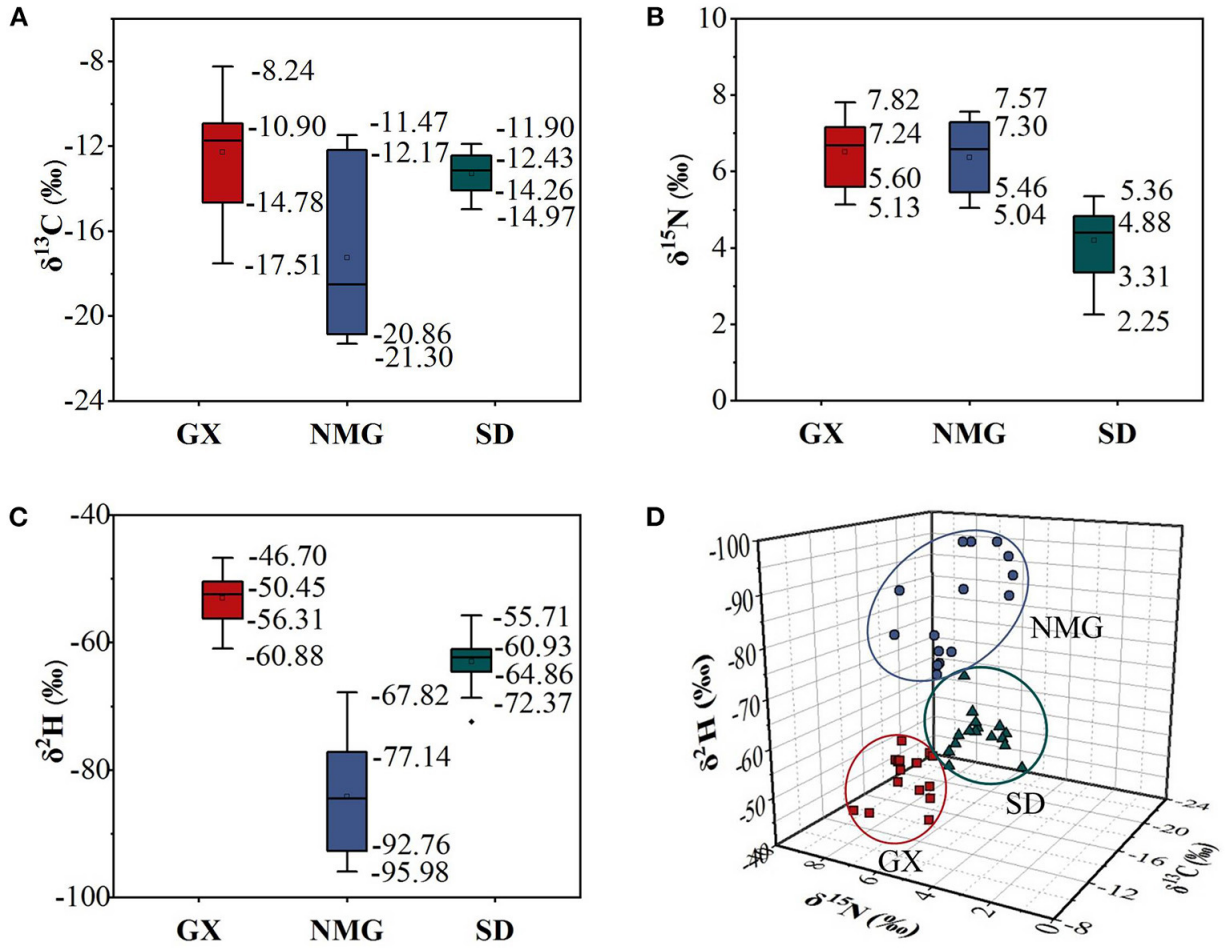
Boxplots of δ^13^C **(A)**, δ^15^N **(B)**, and δ^2^H **(C)** values in gelatin samples from different regions. **(D)** Three-dimensional (3D) scatter plots of δ^13^C, δ^15^N, and δ^2^H in three regions.

Three stable isotope ratios (δ^13^C, δ^15^N, and δ^2^H) and the distribution characteristics of gelatin samples from the three different regions in China are given in Table 2 and Figure 4. According to the results of the ANOVA and three-dimensional (3D) scatter plot of δ^13^C, δ^15^N, and δ^2^H in the three regions, the stable isotope ratios are significantly different (*P* < 0.05) in gelatin samples from the regions.

**Table 2:** A comparison of the mean δ^13^C, δ^15^N, and δ^2^H values and standard deviations of the gelatin samples from three regions.

**Region**	**GX**	**NMG**	**SD**
δ^13^C (‰)	−12.27 ± 2.63a	−17.25 ± 4.04b	−13.27 ± 0.98a
δ^15^N (‰)	6.52 ± 0.88a	6.38 ± 0.91a	4.20 ± 0.91b
δ^2^H (‰)	−52.98 ± 3.90a	−84.19 ± 8.76c	−62.94 ± 3.97b

Different letters indicate significant differences at a p-value of < 0.05.

The δ^13^C of animal products is primarily related to animal feed, particularly the ratio of C_3_ or C_4_ plants (29). In this study, NMG has the lowest δ^13^C (−17.25 ± 4.04‰) value in gelatin samples compared to GX (−12.27 ± 2.63‰) and SD (−13.27 ± 0.98‰). In a previous report, the δ^13^C values of milk samples from Inner Mongolia were also significantly lower than those from Tianjin, Hebei, and Jiangsu (19). The two substances show a similar tendency. This may be because Inner Mongolia has large areas of C_3_ pasture available as the primary fodder source. Another reason for the difference in δ^13^C values between the two may be because of species differences. Guangxi is located in southwest China, which is suitable for C_3_ and C_4_ pasture growing. The cattle from Guangxi are mainly fed C_3_ and C_4_ pasture, but C_4_ pasture is the dominant type. Shandong is located in Eastern China, where wheat and maize are the main products. In the majority of feed given to the cattle, there is a mixture of wheat and maize, with a high proportion of maize. Some studies showed that animals fed C_3_ plants (e.g., wheat) have lower δ^13^C in their tissues than those fed C_4_ plants (e.g., maize) (36). Thus, the δ^13^C values in gelatin samples from NMG are significantly lower than those in the other two regions.

The δ^15^N in animal products is closely linked to the diet consumed by the animals and the regions of their habitat (37). In the present study, the δ^15^N values in gelatin samples from SD (4.20 ± 0.91‰) are significantly lower than those in NMG (6.38 ± 0.91‰) and GX (6.52 ± 0.88‰). This may be due to the cattle from SD being mainly fed maize but the cattle from NMG and GX being mainly fed pasture. During the growing process, maize uses more chemical fertilizers, while pasture uses more organic fertilizers, which are then transferred to the cattle through feed. Compared to organic fertilizers, chemical fertilizers are depleted in δ^15^N (38). Therefore, the δ^15^N values of gelatin in SD are lower than those of the other two regions.

The δ^2^H values of animal products are affected by drinking water and animal feed, which are related to latitude, altitude, and distance from the sea (28). In our study, the δ^2^H values of gelatin are −84.19 ± 8.76‰ in NMG, −62.94 ± 3.97‰ in SD, and −52.98 ± 3.90‰ in GX, indicating a significant difference. Jiang et al. also found that the δ^2^H values of bone samples from Inner Mongolia, Shandon, and Guangxi were in the order of NMG <SD <GX (26). Similar trends appear for both substances. This may be due to NMG having the highest altitude and GX having the lowest altitude among the three regions. The δ^2^H values decrease with increasing latitude, altitude, and distance from the sea, so the δ^2^H values are in the order of NMG <SD <GX.

A significant difference can be noticed in the δ^13^C, δ^15^N, and δ^2^H values in gelatin samples from different regions. Therefore, δ^13^C, δ^15^N, and δ^2^H can be good indicators for the traceability of gelatin.

### 3.4. Difference between gelatin and bone samples

All the data on bone samples are gathered from an article published by our research group (26). Table 3 shows the mean δ^13^C, δ^15^N, and δ^2^H values and standard deviations in the gelatin samples and bone raw materials from different regions. In this study, the δ^13^C values in gelatin samples are slightly higher than those in bone samples, indicating that δ^13^C gets enriched during processing from bone to gelatin, which may be related to the acidification effect. The δ^13^C values were lower when acidification eliminated the ^13^C-enriched carbonate. The δ^15^N values showed no significant variations. In a previous study, Tomaszewicz et al. found that acidification had a minimal effect on the δ^13^C values, reducing marine turtle bone samples by <1‰, and acidification did not affect the δ^15^N values of the milled bone powder, which shows trends similar to the results of our study (39). The δ^2^H values show significant differences, suggesting that the δ^2^H values are significantly affected by processing. Because of the complexity of processing, this aspect needs to be further studied.

**Table 3:** A comparison of the mean δ^13^C, δ^15^N, and δ^2^H values and standard deviations in gelatin samples and bone raw materials from different regions.

**Sample**		**δ^13^C (‰)**	**Δ^13^C**	**δ^15^N (‰)**	**Δ^15^N**	**δ^2^H (‰)**	**Δ^2^H**
GX	Bone	−13.35 ± 2.54		6.08 ± 0.89		−61.09 ± 5.95	
	Gelatin	−12.27 ± 2.63	1.08	6.52 ± 0.88	0.44	−52.98 ± 3.90	8.11
NMG	Bone	−17.75 ± 3.71		5.96 ± 0.90		−82.86 ± 6.49	
	Gelatin	−17.25 ± 4.04	0.50	6.38 ± 0.91	0.42	−84.19 ± 8.76	−1.33
SD	Bone	−13.34 ± 0.94		3.60 ± 0.81		−72.87 ± 2.40	
	Gelatin	−13.27 ± 0.98	0.07	4.20 ± 0.91	0.60	−62.94 ± 3.97	9.93

The symbol Δ represents the difference in δ^13^C, δ^15^N, and δ^2^H values between the gelatin samples and bone raw materials.

**Figure 5:**
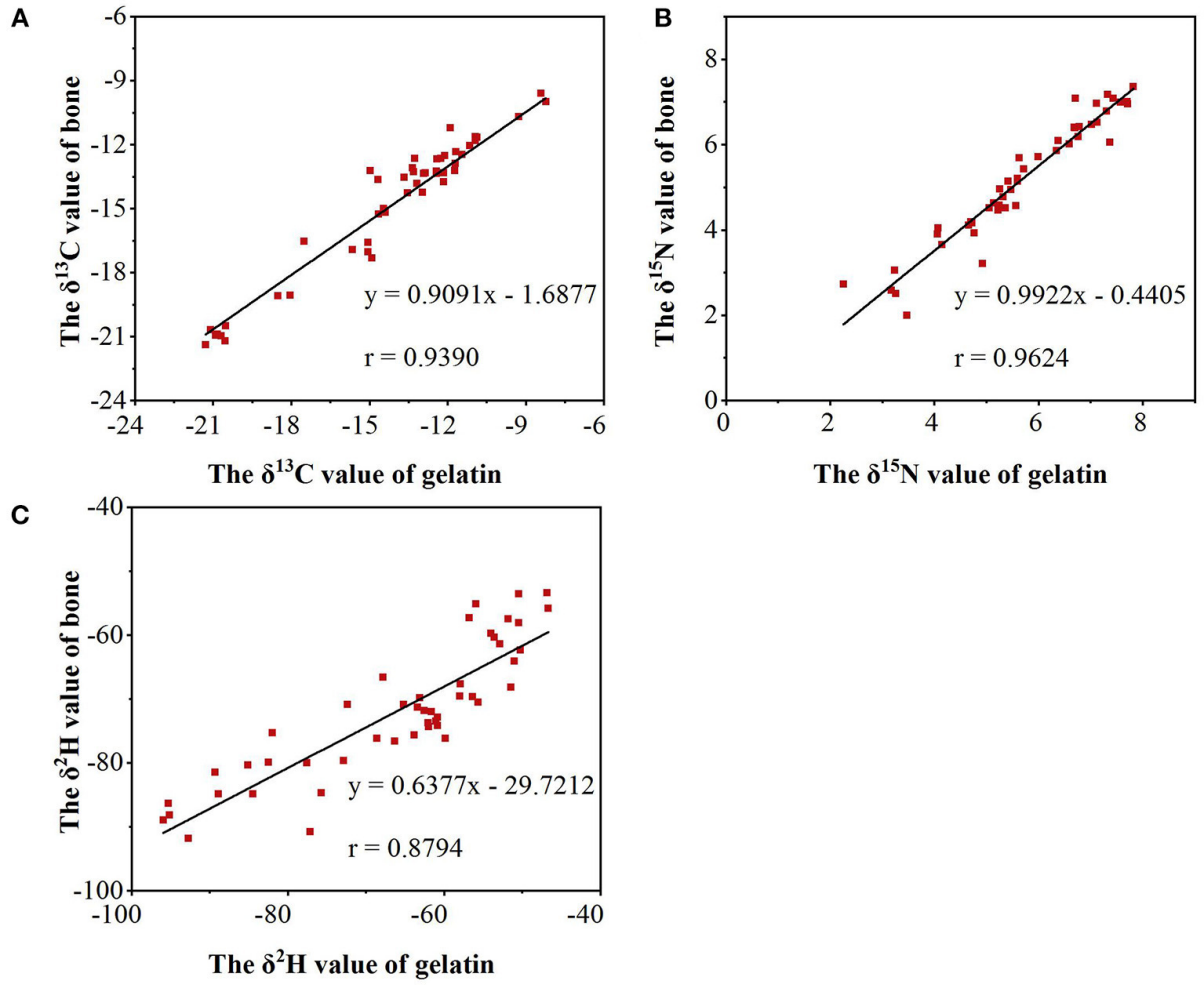
The correlation coefficients of δ^13^C **(A)**, δ^15^N **(B)**, and δ^2^H **(C)** values between the gelatin samples and bone raw materials.

In summary, differences were found in δ^13^C, δ^15^N, and δ^2^H values during the processing from bone to gelatin. However, compared with the differences in δ^13^C, δ^15^N, and δ^2^H values of gelatin from different origins, the fractionation effect caused by processing is not sufficient to influence the identification of gelatin samples from different origins. Meanwhile, a significant correlation was observed among δ^13^C, δ^15^N, and δ^2^H values between the gelatin and bone samples. As shown in Figure 5, the correlation coefficients of δ^13^C, δ^15^N, and δ^2^H values between the gelatin samples and bone raw materials are 0.9390, 0.9624, and 0.8794, respectively (*P* < 0.01). Combined with Table 2, it indicates that δ^13^C, δ^15^N, and δ^2^H can be used as good indicators for gelatin origin traceability.

### 3.5. Discriminant analysis

As single indicators, δ^13^C, δ^15^N, and δ^2^H were not sufficient to identify gelatin samples from different origins; thus, the combination of these three indicators was used to improve the rate of correct classification. As shown in Table 4, the classification model was established using the LDA. Discrimination accuracies of the original classification and cross-validation of gelatin are 97.9 and 95.7%, respectively. Only a few samples from GX and NMG that are misclassified by origin are available. This misclassification may have occurred because the cattle fed on pasture from GX and NMG have a similar feed. These results indicate that the classification model could differentiate gelatin from different regions.

**Table 4:** Discriminant accuracies of the original classification and cross-validation of gelatin samples from different regions.

	**Predicted group membership**					
			**GX**	**NMG**	**SD**	**Total**
Original classification	Number	GX	16	0	0	16
		NMG	1	14	0	15
		SD	0	0	16	16
	%		100.0	93.3	100.0	97.9
Cross-validation	Number	GX	16	0	0	16
		NMG	1	14	0	15
		SD	1	0	15	16
	%		100.0	93.3	93.8	95.7

## 4. Conclusion

This study investigated the feasibility of using stable isotope ratio analysis combined with chemometric analysis to identify the origins of gelatin. The results show that stable isotopes (δ^13^C, δ^15^N, and δ^2^H) could be used as effective origin indicators for gelatin traceability, and the classification model also shows high discriminant ability. Therefore, stable isotope ratio analysis coupled with chemometric analysis can be used as an effective method for gelatin traceability. Furthermore, the results of this study may provide technical support for the quality and safety control of gelatin.

## Data availability statement

The raw data supporting the conclusions of this article will be made available by the authors, without undue reservation.

## Author contributions

SL: methodology, investigation, validation, and writing. DJ: methodology, formal analysis, reviewing, and editing. JL: software and formal analysis. YM, JY, and LD: formal analysis support. YX: supervision, reviewing, and editing. YQ: supervision, funding acquisition, review, and editing. All authors contributed to the article and approved the submitted version.
